# A chimeric IgE that mimics IgE from patients allergic to acid-hydrolyzed wheat proteins is a novel tool for *in vitro* allergenicity assessment of functionalized glutens

**DOI:** 10.1371/journal.pone.0187415

**Published:** 2017-11-08

**Authors:** Olivier Tranquet, Jean-Charles Gaudin, Sarita Patil, Johanna Steinbrecher, Kayoko Matsunaga, Reiko Teshima, Shinobu Sakai, Colette Larré, Sandra Denery-Papini

**Affiliations:** 1 UR 1268 Biopolymers Interactions Assemblies, INRA, Nantes, France; 2 Center for Immunology and Inflammatory Diseases, Massachusetts General Hospital and Harvard Medical School, Boston, Massachusetts, United States of America; 3 Department of Integrative Medical Science for Allergic Disease, Fujita Health University School of Medicine, Toyoake, Japan; 4 National Institute of Health Sciences, Tokyo, Japan; Forschungszentrum Borstel Leibniz-Zentrum fur Medizin und Biowissenschaften, GERMANY

## Abstract

**Background:**

Acid-hydrolyzed wheat proteins (acid-HWPs) have been shown to provoke severe allergic reactions in Europe and Japan that are distinct from classical wheat allergies. Acid-HWPs were shown to contain neo-epitopes induced by the deamidation of gluten proteins. However, products with variable rates of deamidation can be found.

**Objectives:**

In this work, we studied the effect of the extent of wheat proteins deamidation on its allergenicity. A recombinant chimeric IgE was produced and compared to patients’ IgE for its capacity to assess the IgE-mediated triggering potential of acid-HWPs.

**Methods:**

Sera from acid-HWP allergic patients were analyzed via ELISA and a functional basophil assay for their IgE reactivity to wheat proteins with different deamidation levels. A chimeric mouse/human IgE (chIgE-DG1) specific for the main neo-epitope, QPEEPFPE, involved in allergy to acid-HWPs was characterized with respect to its functionality and its reactivity compared to that of patients’ IgE.

**Results:**

Acid-HWPs with medium (30%) and high (50–60%) deamidation levels displayed a markedly stronger IgE binding and capacity to activate basophils than those of samples with weak (15%) deamidation levels. The monoclonal chIgE-DG1 allowed basophil degranulation in the presence of deamidated wheat proteins. ChIgE-DG1 was found to mimic patients’ IgE reactivity and displayed the same ability to rank acid-HWP products in a degranulation assay.

**Conclusion:**

Increasing the deamidation level of products from 15% to 60% resulted in an approximately 2-fold increase in their antigenicity and a 100-fold increase in their eliciting potential. The chimeric ChIgE-DG1 may be a useful tool to evaluate functionalized glutens for their allergenic potential. By mimicking patient sera reactivity, chIgE-DG1 also provided data on the patients' IgE repertoire and on the functionality of certain repeated epitopes in gluten proteins.

## Introduction

A large proportion of food allergies are IgE mediated. In these patients, the immune system is unbalanced towards a Th2 profile and allergens induce the production of immunoglobulins E (IgE), which bind to high-affinity IgE receptors (FcεRI) expressed on effector cells (e.g., mast cells and basophils). Subsequent crosslinking of IgE by allergens induces clustering of FcεRI and subsequent effector cell degranulation with the release of mediators such as histamine, leukotrienes and cytokines, resulting in allergic symptoms.

Wheat proteins, of which at least 80% are gluten proteins, have been shown to trigger IgE-mediated food, respiratory and contact allergies [1,2]. The group containing gluten proteins is composed of approximately 100 proteins, which can be classified into 2 groups: monomeric proteins (the gliadins) and polymeric proteins (the glutenin subunits). Gluten proteins contain large repeat domains composed of homologous and repetitive sequences of 6 to 8 amino acids rich in proline and glutamine [3]. Beyond their traditional uses in bakery and pastry, wheat proteins can be transformed by enzymatic or chemical hydrolysis to increase their water solubility and confer new foaming and emulsifying functionalities [4,5]. One of these processes, acid hydrolysis of wheat proteins via heating in acidic conditions, is conducted at the industrial scale to produce ingredients for food, feed and cosmetics. During such mild chemical treatment, gluten proteolysis is generally limited, but deamidation, which leads to the conversion of glutamines into glutamic acids, occurs to a larger extent.

Beginning in the 2000s, case reports have described allergic reactions to hydrolyzed wheat proteins (HWPs) in several European countries [6–8]. In 2012, our group described 15 patients with allergy to acid-HWPs. All of them had experienced severe allergic symptoms (anaphylaxis, exercise induced anaphylaxis or urticaria) after ingestion of food products containing HWPs. The patients were sensitized to neo-epitopes generated by acid hydrolysis of gluten. Allergy to acid-HWPs was therefore considered a new form of allergy. From 2002 to 2014, 25 severe cases were reported by the French Allergy Vigilance Network (http://www.allergyvigilance.org/). More dramatically, in Japan, a facial soap containing the acid-HWP Glupearl19S^®^ (GP19S) was involved in more than 2000 cases of severe allergic reactions [9]. The sensitization mechanism probably occurred through contact via percutaneous or rhinoconjunctival routes over several months, after which the patients developed anaphylaxis or exercise induced anaphylaxis when ingesting classical wheat-based products [10].

The same IgE-binding consensus peptide, QPQQPFPQ, was identified for the French and Japanese patients [11,12] and corresponds to a repeat motif of γ and ω2 gliadins. The neo-epitope QPEEPFPE generated by deamidation of the QPQQPFPQ motif was found to be an immune-dominant epitope in the French patients. In the Japanese patients, peptides containing the deamidated PEEPFP sequence were also more reactive than the native epitope.

During acid hydrolysis of gluten proteins, deamidation does not occur on all glutamines. Acid-HWPs undergo variable rates of deamidation and hydrolysis depending on treatment intensity (pH, temperature, duration) [13,14], resulting in ingredients with different functionalities [5,15]. Monoclonal antibodies (mAbs) have been produced to detect deamidated gluten for food safety [13,16]. Tranquet et al. produced the mouse antibody INRA-DG1 (mDG1) raised against the neo-epitope QPEEPFPE, which is capable of discriminating between deamidated and native gluten sequences. Using this mAb together with mAb R5 specific for native sequences [17,18], Tranquet et al. revealed the heterogeneity of the deamidation level of industrially produced HWP [13]. However, nothing is known about the allergenicity of such products as a function of their deamidation level.

The potency of acid-HWP products to trigger reactions, i.e., their capacity to activate allergic effector cells in the presence of allergen-specific IgE, can be addressed with basophils and IgE. Humanized rat basophilic leukemia (RBL) cell lines, which express at least the α-chain of human FcεRI, can be used as a functional assay in the presence of human sera [19,20]. Indeed, this model was previously applied to demonstrate the capacity of acid-HWPs to trigger allergic reactions in French and Japanese patients [11,14]. However, sera from patients allergic to acid-HWPs are available in very limited amounts to analyze the eliciting capacities of functionalized glutens.

Recombinant monoclonal IgEs have been used as serum substitutes for studying the molecular properties of allergens and IgE/allergen interactions [21,22]. In particular, chimeric mouse/human IgEs have been produced to investigate on model allergens how IgE properties and epitope repertoires affect effector cell activation [23,24]. Recently, a combination of 6 recombinant chimeric IgEs specific for β-lactoglobulin was generated to assess the allergenic potential of whey hydrolysates [25].

The present work aimed to clarify how the extent of deamidation affects allergenicity by analyzing the reactivity of IgE from acid-HWP-allergic patients towards HWP products showing various deamidation levels. A chimeric mouse/human IgE directed to the neo-epitope, QPEEPFPE, was also studied for its capacity to be used in assessing the triggering potential of these products.

## Materials and methods

### Ethics statement

Human sera were collected from 2001 to 2014. All sera were collected into three distinct clinical trials corresponding to 3 successive research projects on the characterization of wheat allergens before their inclusion into our bio-collection (DC-2008-809). The first clinical trial was funded by the INRA call “Food, quality and Security: Identification of wheat allergenic proteins”. It was approved by the Ethical Committee of the Cochin hospital (Paris, France). The second was funded by the INRA Food Research Program: Study of feasibility of wheat based products for some wheat allergic patients. It was approved by the Ethical Committee of Biomedical Research from Lorraine and by the Health Ministry (DGS2007-0066). The third was funded by the National Research Agency: PREDEXPITOPE (prediction and experimental validation of epitopes of wheat allergens). It was approved by the Ethical Committee of Ile de France III and AFSSAPS (authorization number 2008-A01565–50). These 3 clinical trials received ethical approvals for serum collection and their use for research purposes and written consents were obtained from the patients or their parents.

### Native and deamidated wheat proteins

Laboratory and industrial samples of native and deamidated gliadins and glutens were used in this study. Native gluten and native gliadins were previously prepared from wheat flour (c.v. recital) according to Battais et al. [26]. Briefly flour was defatted and gluten was separated from starch by extensive washing of the dough by water. The gliadin fraction was isolated from the gluten with 70% ethanol (v/v). Three fractions of deamidated gliadins with deamidation rates of 15%, 35% and 48% (referred to as D-GLIA 15, 35 and 48) have been produced by acid hydrolysis of gliadins and characterized in a previous study [13]. Briefly, Gliadins (10 mg) were solubilized in 1 mL of 50% (v/v) ethanol, 0.1 N hydrochloric acid, and heated at 90°C for 40, 90, and 120 min. Deamidated Gluten (D-Gluten) was prepared by acid hydrolysis of gluten according to Gourbeyre et al. [27]: Gluten (20 mg) was solubilized in 1 mL of 0.1 N hydrochloric acid, and heated at 90°C for 1h. The reaction was stopped by neutralization with 1 mL 0.1 N NaOH. Solutions were dialyzed against water and freeze-dried.

Industrial samples of HWP (HWP1 to HWP4 and GP19S) have been commercialized for food or cosmetic applications in Europe and in Japan and were previously described in former papers [7,11,13,14,28]. HWP1, 2 and 4 and GP19S have been prepared by acid hydrolysis; no data was available for HWP3. The deamidation rates were only specified for HPW1 and 2 (respectively 20–30% and 60%). The deamidation level of other products was estimated by a competitive ELISA using mAb mDG1 as described by Tranquet et al. The proteolysis extent of samples was analyzed by SDS-PAGE.

### Subjects

Sera were obtained from nine subjects with food allergy to acid-HWP (S1 Table). The patients experienced symptoms of urticaria, exercise-induced anaphylaxis or anaphylactic shock. Patients 30 and 34 also presented contact urticaria with cosmetics containing HWP. One of these patients (n°34) presented also symptoms of exercise-induced anaphylaxis due to wheat. The other patients were tolerant to native wheat proteins. Allergy to acid-HWP was established on the basis of the clinical history and/or by a positive double-blind placebo-controlled food challenge. Sensitization to deamidated or native wheat proteins was assessed by skin prick tests to wheat flour, gluten (ALK-Abello), and deamidated gluten (ALK-Abello isolate) or by specific IgE determination in fluorimetric ELISA (F-ELISA) using native fractions (gliadins, ω5-gliadins, LTP, albumins/globulins) and deamidated gluten (D-Gluten). Six of the sera (#30, 34, 285, 299, 352 and 390) had been previously used in Denery et al. (2012). A serum from one patient suffering from food allergy to wheat with atopic dermatitis was used as control (#1274).

### Fluorimetric ELISA

Sera were analyzed for specific IgE by F-ELISA on white NuncMaxiSorp 384 well microtiter plates (Fischer Scientific, France) with a Biomek NXP automated workstation (BEKMANCoulter, France) as described [29]. Antigens were solubilized at 1 mg/mL by continuous agitation overnight at room temperature. Native gliadins were solubilized in 50% ethanol and D-GLIA 15, 35 and 48 were solubilized in phosphate buffered saline (PBS: 137 mM NaCl, 3 mM KCl, 8 mM Na_2_HPO_4_, 1 mM KH_2_PO_4_; pH 7.2). Gluten and Acid-HWP were solubilized in 0.1 M carbonate buffer (pH 9.6) with 0.1% SDS and 0.2% 2-mercaptoethanol. All antigens were then diluted in 0.1M carbonate buffer (pH9.6) at 5μg/mL for coating. Human sera (diluted 1:10) and chIgE-DG1 (1:1000) were analyzed in triplicate. Concentrations in specific IgE were calculated by reference to a standard curve included in each microtiter plate (from 160 ng/ mL to 0.07 ng/mL of standard human IgE—2nd WHO international reference NIBSC 75/502). The standard curve was fit using four-parameter logistic regression. For each serum, results were normalized in comparison to the laboratory D-gluten sample by determining the ratios of antigen-specific IgE concentrations to the D-Gluten-specific IgE concentration. To determine differences in antigen recognition, matched ratios per serum were analyzed by repeated-measures one-way ANOVA and subsequent Bonferroni’s multiple-comparisons test (p<0.05) using GRAPHPAD PRISM version 5.02 (GraphPad Software Inc., USA).

### Construction of mouse/human IgE-DG1 heavy and light chain plasmids

Mab mDG1 (Tranquet, Lupi, Echasserieau-Laporte, et al. 2015) was raised in mouse against the peptide LQPEEPFPEQC containing a dominant IgE-binding epitope for patients allergic to acid-HWP. Variable domains of heavy and light chains (VH and VL) of mDG1 were cloned in pMD18 and sequenced (Genscript, Piscataway, USA). In order to express in mammalian cells the chimeric mouse/human IgE specific for this deamidated epitope we used two plasmids: pLNOH-2IgE and pLNOK (Norderhaug et al. 1997; Christensen et al. 2008). pLNOH and pLNOK had been designed to express IgG3 heavy chain and kappa light chain (Norderhaug et al. 1997), pLNOH was further modified to give pLNOH-2IgE in order to express IgE heavy chain (Christensen et al. 2008). pLNOH and pLNOK DNA were kindly provided by Pr Lund (ALK-Abello, Horsholm, Denmark). cDNA of mDG1 variable domains VH and VL were cloned in pMD18 and amplified by PCR with VH (5’VH primer: TGC ATT CCG ATG TGC AGC TTC AGG; 3’ VH primer: CGT ACG ACT CAC CTG AGG AGA CGG TGA CCG T) and VK (5’VK primer TGC ATT CCG ATG TTG TGA TGA CCC AA; 3’VK primer CGT ACG ACT CAC GTT TGA TTT CCA GCT TGG TG) primer pairs respectively. These primers contained bsmI (bold) or bsiWI (underlined) restriction sites to allow cloning of the VH PCR product into plasmid pLNOH-2IgE and of the VK PCR product into plasmid pLNOK. In a first step, PCR products were ligated into pGEMTeasy (Promega, France) to facilitate digestion with bsmI and bsiWI and subcloning onto pLNOH-2IgE and pLNOK.

### Expression of chimeric IgE-DG1

Full-length recombinant chimeric human IgE (chIgE-DG1) containing the mDG1 paratope was expressed by transient co-transfection of HEK293 cells (ATCC^®^ CRL-1573) with pLNOH2IgE-DG1VH and pLNOK-DG1VL plasmids. To this end, adherent HEK293 cells were cultured at 5% CO_2_ and 37°C in DMEM containing 4.5 g/L L-glucose (ThermoFisher) and supplemented with 10% decomplemented FBS (Lonza, France), 4 mM L-glutamine, 100 U/mL penicillin (Lonza, France) and 100 μg/mL streptomycin (Lonza, France). The day before transfection, 3 x 10^5^ cells were seeded into 24-well culture plates and grown for 24 h in 293 FreeStyle^™^ expression medium (ThermoFisher). Co-transfection with 1 μg of each heavy and light chain-encoding plasmid was performed using 293fectin^™^ (ThermoFisher, France) and Opti-MEM (ThermoFisher, France) according to the manufacturer’s instructions. ChIgE-DG1 containing supernatant was collected 4 days after transfection and cleared by centrifugation for 10 min at 2500 g before 0.22 μm filtration and dialysis against PBS by ultracentrifugation on an Amicon^®^ 50 kDa MWCO Ultra cell Unit (Millipore, France) and stored at -20°C.

ChIgE-DG1 expression was controlled by Western blotting. Ten microliters of supernatant was loaded on a 12% precast gel (Bio-Rad, France) and separated by SDS-PAGE. Proteins were transferred onto a 0.22 μm nitrocellulose membrane (Bio-Rad, France) using the semi-dry transfer system (TransBlot Turbo, Bio-Rad). After saturation with PBS containing 4% BSA (SIGMA, France), the membrane was incubated with peroxidase-conjugated anti-human IgE (1/3000, art. n° 9160–05, SouthernBiotech, USA) or peroxidase-conjugated anti-human kappa light chains (1/3000, art. N°GtxHu-084-DHRPX, ImmunoReagents, USA) diluted in PBS containing 0.1% BSA. Detection was performed using ECL reagent (Western Bright Quantum, Advansta, USA) and a cooled digital camera (LAS3000, Fuji).

The activity and specificity of chIgE-DG1 were controlled and compared with those of mDG1 via indirect ELISA on native or deamidated gliadins coated at 5 μg/mL. ChIgE-DG1 was revealed with peroxidase-conjugated anti-human IgE, while mDG1 was revealed with peroxidase-conjugated goat anti-mouse IgG (1/3000, art. n° 170–6516, Bio-Rad).

### Basophil activation assays

Degranulation assays were performed either with stripped human basophils as previously described [30] or with the humanized rat basophil cell line RBL-SX38 (clone P4B7), which expresses the α, β and γ chains of the human FcεRI [19] and was kindly provided by Pr. Kinet (Harvard Medical School, New York, USA).

For the stripped basophil assay, buffy coats were obtained from healthy, de-identified blood donors at the Massachusetts General Hospital Blood Donor Center (Boston, MA). The study was approved by the Institutional Review Board of Partners Healthcare (Boston, MA). Peripheral blood mononuclear cells (PBMCs) were isolated by Ficoll centrifugation. After washing, IgEs were stripped from the cell surface with a lactic acid buffer as previously described [31]. The cells were then passively sensitized by incubation at 37°C for 60 min with chIgE-DG1 at 2 μg/mL in RPMI/0.5% BSA. After washing, sensitized PBMCs were stimulated with D-Gluten (1ng/mL- 100μg/mL, diluted from a stock solution at 1 mg/mL in 0.1 M carbonate buffer pH 9.6 with 0.1% SDS and 0.2% 2-mercaptoethanol) in RPMI/BSA containing 2 μg/mL IL-3 (Shenandoah Biotechnology rH-IL3 100–30). Anti-IgE antibody (5 μg/mL, Bethyl Laboratories A80-109A) or RPMI/BSA/IL-3 alone was used as a control. Stimulation of 2 x 10^6^ cells per condition was performed for 30 min at 37°C. The reaction was stopped with EDTA, and cells were stained by incubation at 4°C with labeled anti-CD123-phycoerythrin-cyanin5 (BioLegend, San Diego, Calif), CD63-fluorescein isothiocyanate (eBioscience, San Diego, Calif), and CD203c-phycoerythrin-cyanin 7 (BioLegend, San Diego, Calif) followed by flow cytometric analysis. Basophils were gated as side scatter–low, CD123^+^, and CD203c^+^ cells. Cells were measured with a FACS LSRII flow cytometer (BD Biosciences), and data were analyzed using BD FACS Diva software (BD Biosciences). CD63^++^ basophils were determined after stimulation with D-gluten and after stimulation with anti-IgE. Basophil activation was expressed as the percentage of CD63^++^ basophils induced by D-gluten to CD63^++^ basophils induced by the anti-IgE antibody.

For the humanized RBL assay, sera were decomplemented by heating for 45 min at 56°C in the presence of 4 M glucose to reduce cytotoxicity [32]. RBL-SX38 cells at 1.2x10^4^ or 2.5x10^4^ cells/well in 96-well flat-bottom culture plates were grown for 24 h before sensitization with patient sera (diluted 1:25) or with chIgE-DG1 (100 ng/mL) for 24 h. Sensitized RBL-SX38 cells were washed 3 times with PBS before stimulation with either mouse anti-human IgE (0.5 μg/mL; Le27 clone, NBS-c BioScience, Vienne, Austria) or with antigens (0.1 ng/mL to 10 μg/mL) diluted in Tyrode buffer with 50% D_2_O. β-hexosaminidase release induced by the crosslinking of the FcεRI was measured with pNAG (art. N° N9376, SIGMA) as previously described [20]. For each serum or chIgE-DG1, spontaneous release was determined with unstimulated cells and used as the background value. The β-hexosaminidase release induced by the samples was expressed as the percentage of the release observed with anti-human IgE antibodies (set as 100% degranulation). Curves presenting the percentage of degranulation as a function of allergen concentration were fit using four-parameter logistic regression. Allergen concentrations corresponding to 50% of the highest release (EC_50_) were then determined using GraphPad Prism 5.02 for Windows (GraphPad Software Inc., La Jolla, CA, USA). The EC_50_ values of different samples were compared by two-way ANOVA followed by Bonferroni’s multiple-comparisons test.

For inhibition experiments, cells were sensitized with 6 patients' sera and mDG1 [13] was used as an inhibitor of acid-HWP-induced degranulation. Acid-HWP samples (HWP-2 and GP19S) at 200 ng/mL were incubated with mDG1 at 20 μg/mL for 1 h at 37°C in 2X Tyrode buffer before dilution in D_2_O (v/v) and stimulation of sensitized RBL-SX38 cells. Data were analyzed with a Wilcoxon matched-pairs signed rank test for HWP2 or GP19S (*: p ≤ 0.05).

## Results

### Characterization of industrial HWP and laboratory deamidated wheat proteins

Products with known and unknown levels of deamidation were used in the study. To enable a comparison among them, a competitive ELISA with mAb mDG1 was performed on all samples and the relative abundance of deamidated sequences was estimated using a comparison of their IC_50_ [13](Table 1). Three groups of products were extrapolated from this analysis and were characterized by their extent of deamidation: weak (D-GLIA 15 and HWP4 with deamidation level approximately 15%), medium (D-GLIA 35 and HWP1 with deamidation level approximately 30%) and high (D-GLIA 48, HWP2 and 3 and GP19S with deamidation level approximately 50–60%). The migration patterns of HWP samples in SDS-PAGE suggested that concomitantly with deamidation, strong proteolysis had occurred for HWP3, while it was only moderate for other samples (S1 Fig).

**Table 1:** Relative abundance of deamidated sequences detected by mDG1 in deamidated protein fractions. Six concentrations (6 ng/mL-20 μg/mL) of native or deamidated samples were analyzed with a competitive mDG1 ELISA. For each sample, IC_50_, i.e., the concentration that inhibited half of the maximum binding, was graphically determined.

	HWP1	HWP2	HWP3	HWP4	GP19S	D-GLIA 15	D-GLIA 35	D-GLIA 48
IC competitive ELISA	0.3	0.05	0.1	2	0.04	4	0.5	0.08
% Deamidation	25–30^a^	60^a^	50–60^c^	15^c^	50–60^c^	15^b^	35^b^	48^b^

^a^ data from manufacturer

^b^ data from Tranquet et al. 2015

^c^ percentage of deamidation estimated by comparison to other samples

### Determination of patient IgE reactivity to the various deamidated wheat proteins in ELISA

**Fig 1:**
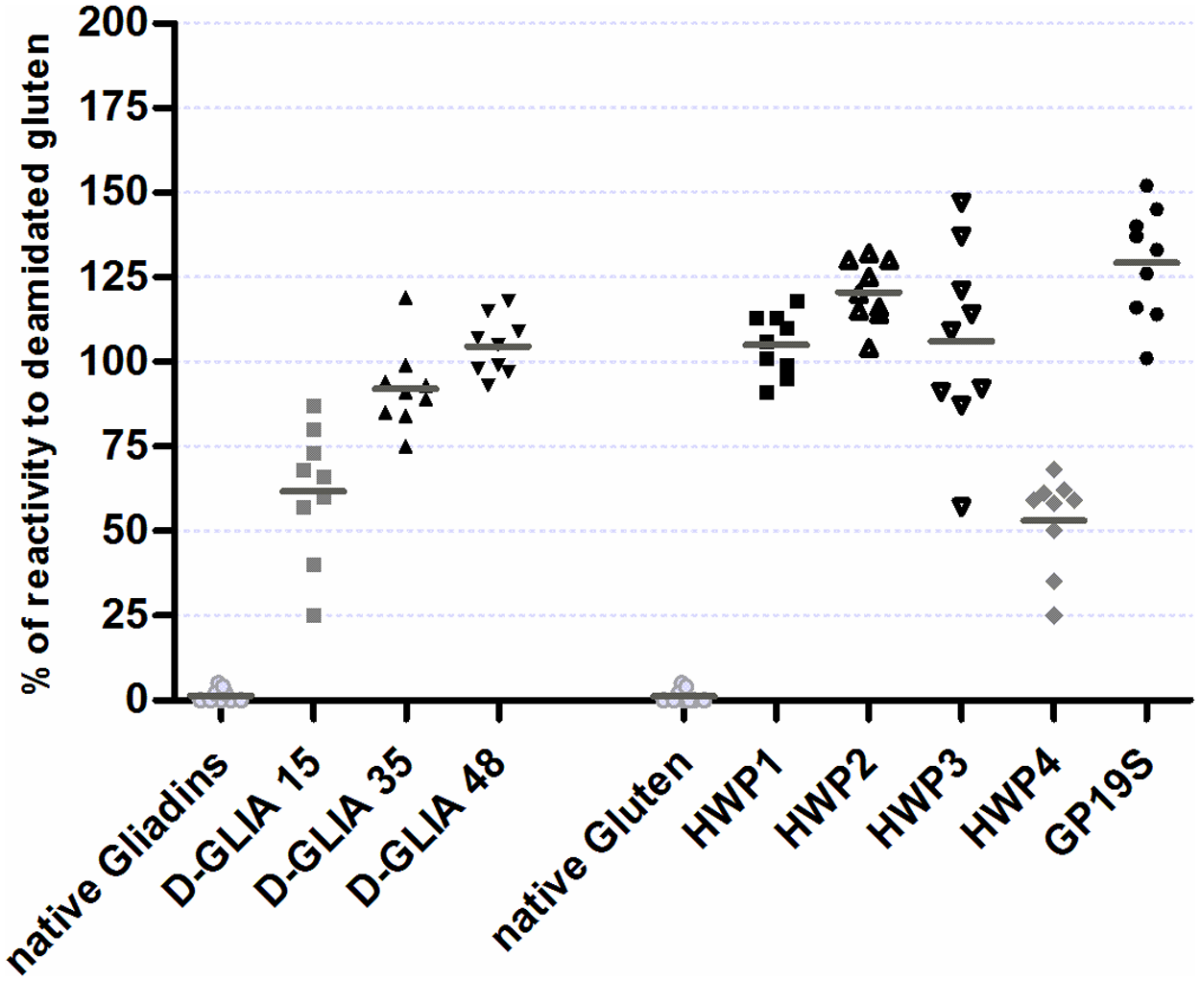
Reactivity of patient IgEs to deamidated wheat proteins with different deamidation levels. Concentrations of patients' specific IgEs for native gliadins, native gluten, deamidated gliadins (D-GLIA15, 35, 48), and gluten (D-Gluten), and industrial samples of HWP (HWP1, 2, 3, 4 and GP19S) were determined by indirect ELISA. The results were normalized by determining the ratios of the specific IgE for each protein fraction to the specific IgE for D-Gluten. Symbols represent means of triplicates. Statistical analysis by Bonferroni’s multiple comparison test of the means of the ratios revealed 3 distinct groups of samples (p<0.001): 1) native gliadins and native gluten (light grey symbols), 2) D-GLIA 15 and HWP4 (dark grey symbols), and 3) samples with medium and high deamidation rates: HWP1, 2 and 3 and GP19S and D-GLIA35 and 48 (black symbols). HWP, hydrolyzed wheat protein. GP19S, Glupearl 19S^®^.

The sera from nine acid-HWP-allergic patients were used for this study. Native and deamidated gliadins, gluten and industrial HWP-specific IgE concentrations were measured by F-ELISA. Patients’ IgEs were almost exclusively directed towards deamidated proteins since they displayed no or very low reactivity to native gliadin or gluten extracts (S2 Table). To compare the reactivity profiles of these patients independently of their total and specific IgE concentrations, which varied considerably from one patient to another, the results were normalized using D-Gluten-specific IgE as an internal reference. Fig 1 shows the ratios of the specific IgE concentration for each sample to the specific IgE concentration for D-Gluten. Statistical analysis of the means of the ratios revealed 3 distinct groups of samples (p<0.001): 1) native gliadins and native gluten, 2) weakly deamidated samples, and 3) samples with medium and high deamidation rates. Indeed, the mean ratios calculated for the weakly deamidated laboratory (D-GLIA 15) and industrial (HWP4) samples were not significantly different from one another, while they were significantly lower than those calculated for the medium and highly deamidated gliadin and HWP samples (HWP1, 2 and 3 and GP19S and D-GLIA35 and 48). Heterogeneity was observed in patients’ IgE binding for some deamidated samples, particularly for D-GLIA15 and HWP4 and HWP3.

### Characterization of patient IgE reactivity to the various deamidated wheat proteins using humanized RBL assay

RBL-SX38 cells were passively sensitized with the sera from patients allergic to acid-HWPs or with a serum from a wheat-allergic patient (#1274) and were stimulated with D-Gluten (from 1 to 1000 ng/mL) (data not shown). Wheat-allergic serum-sensitized RBL cells did not induce any β-hexosaminidase release upon stimulation with any doses of D-Gluten. However, acid-HWP-allergic sera (n = 7 of 9) -sensitized RBL cells resulted in the release of β-hexosaminidase to D-gluten in a dose-dependent manner. Similar EC_50_ values (approximately 10 ng/mL) were found for the 7 sera, although both the concentration and the proportion of specific IgEs to D-gluten of the total IgE varied considerably among the sera (S2 Table).

**Fig 2:**
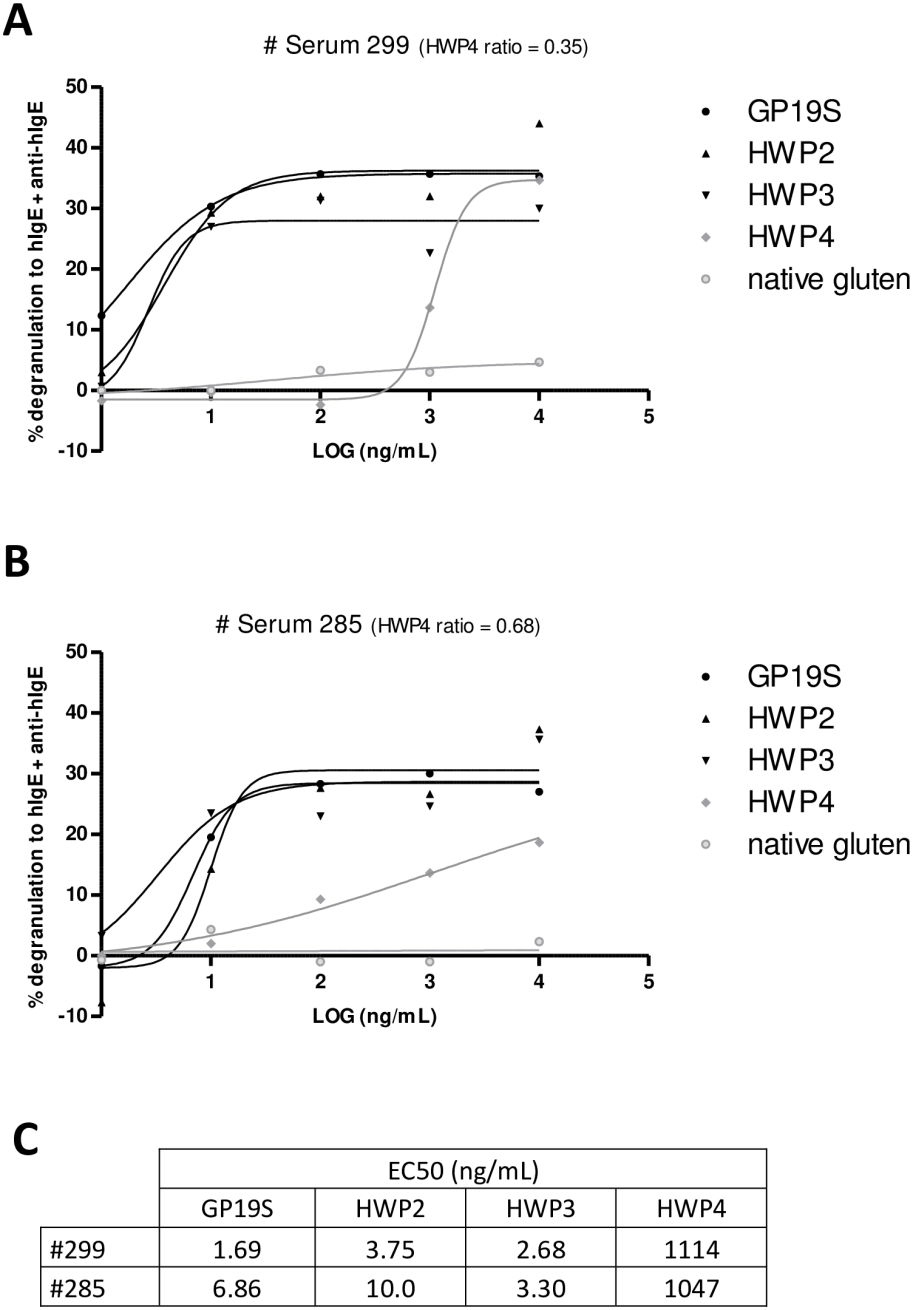
Degranulation induced by different HWPs of RBL-SX38 cells sensitized with sera from 2 HWP-allergic patients. Human FcεRI-expressing RBL cells were passively sensitized with serum #299 (A) or with serum #285 (B) and subsequently activated with anti-human IgE antibody or five concentrations (1 ng/mL to 10 μg/mL) of native gluten, HWP2, 3, 4 or GP19S. The results are expressed as percentages of degranulation compared to anti-human IgE-induced degranulation. Symbols represent means of triplicates. EC_50_ values were determined using GraphPad Prism 5.02for Industrial HWPs (C). HWP, hydrolyzed wheat protein. GP19S, Glupearl 19S^®^.

Two sera (#285 and #299) were used to investigate the capacity of the different HWP samples to induce RBL activation. These 2 sera were selected because they bound differently to weakly deamidated samples in ELISA (ratio of 0.68 versus 0.35 on HWP4 for sera #285 and 299, respectively). Native gluten did not induce cell degranulation (Fig 2). The products with the highest deamidation levels (HWP2, HWP3 or GP19S) induced the most efficient RBL activation, irrespective of the sensitizing serum. Statistical analysis of the EC_50_ revealed that HWP2, HWP3 and GP19S were not significantly different (p<0.05), while HWP4 displayed a lower triggering capacity (p<0.01). These results suggest that patient IgE preferentially binds to highly deamidated products, which possess strong RBL activation abilities.

### Inhibition of RBL degranulation by anti-peptide mDG1

**Fig 3:**
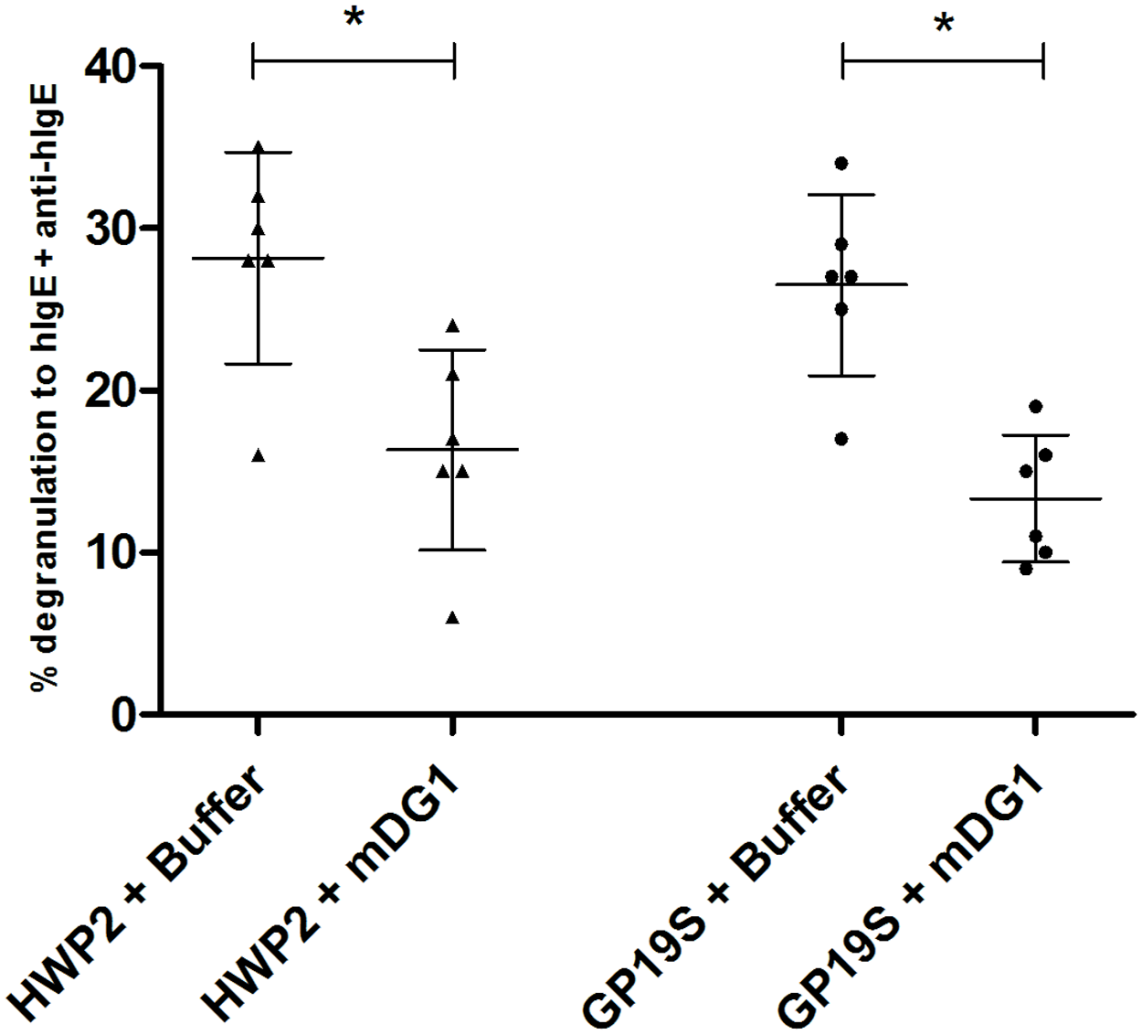
Inhibition of HWPs-induced-degranulation of RBL-SX38 cells by mAb mDG1. Human FcεRI-expressing RBL cells were passively sensitized with 6 sera (#30, 285, 299, 352, 1414, 1649) and subsequently cross-linked with anti-human IgE antibodies, HWP2 (100 ng/mL) or GP19S (100 ng/mL), pre-incubated for 1 h with purified mouse monoclonal antibody mDG1 (20 μg/mL). The results are expressed as percentage of degranulation compared to anti-human IgE-induced degranulation. HWP, hydrolyzed wheat protein. GP19S, Glupearl 19S^®^. Symbols represent means of triplicates. Horizontal bars represent mean, and error bars represent ± SD. Data were analyzed with a Wilcoxon matched-pairs signed rank test for HWP2 or GP19S (*: p ≤ 0.05).

To further explore the contribution of highly deamidated sequences to IgE binding and RBL cell activation, mDG1 was used for inhibition experiments. This mouse mAb specifically binds the highly deamidated sequences E/QPEEPFPE and more weakly to the sequences E/QPQEPFPE [13]. The ability of mDG1 to inhibit HWP2- or GP19S-induced degranulation was investigated with 6 patients’ sera. Both HWP2 and GP19S induced equivalent percentages of degranulation (means 28% and 27%, respectively) of RBL cells in the absence of an inhibitor (Fig 3). Pre-incubation with mDG1 systematically and significantly reduced the percentages of degranulation induced by either HWP2 or GP19S compared with those induced with the buffer alone (p = 0.0340 and p = 0.0355). The mean inhibition of the degranulation by mDG1 was 45% (range 30–63%) and 50% (33–72%) for HWP2 and GP19S, respectively.

### Generation and characterization of a chimeric mouse/human IgE DG1 specific for acid-HWPs

**Fig 4:**
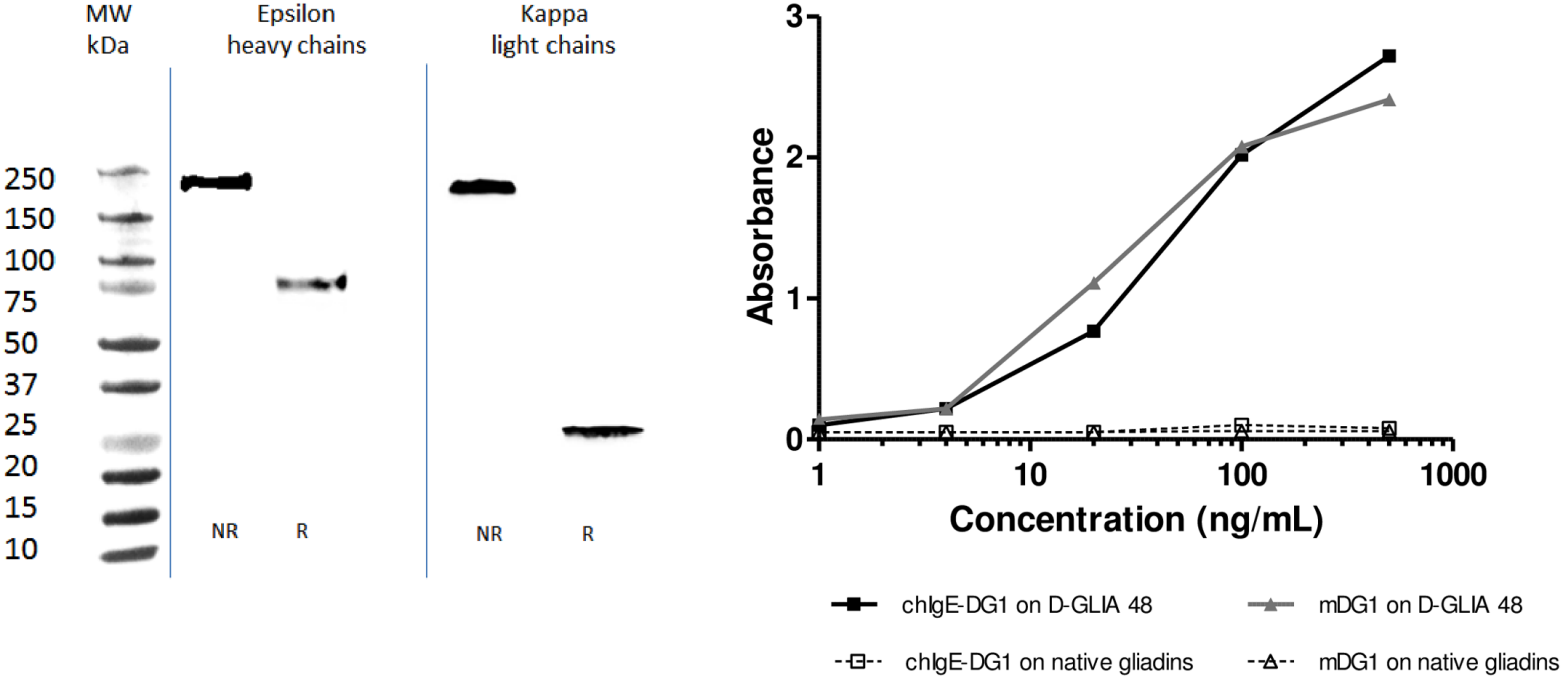
Characterization of chimeric mouse/human IgE-DG1. Chimeric mouse/human IgE-DG1 was transiently expressed in HEK293 cells, and the IgE concentrations were determined by ELISA. A. Non-reduced (NR) or reduced (R) proteins from the supernatant of transfected HEK293 cells were separated by SDS-PAGE (12%) and transferred onto nitrocellulose membrane. IgE heavy and kappa light chains were detected by peroxidase-conjugated anti-human epsilon heavy chains or by anti-human kappa light chains, respectively, prior to chemiluminescent visualization. B. The activity and specificity of chimeric IgE-DG1 and its mouse monoclonal IgG counterpart (mDG1) were compared by indirect ELISA. Native gliadin- or deamidated gliadin (D-GLIA 48) -coated ELISA plates were incubated with indicated concentrations of chimeric IgE-DG1 or mDG1.

Since the highly deamidated sequences bound by mDG1 notably contributed to RBL cell degranulation, this mAb was used to produce a chimeric mouse/human recombinant IgE. cDNAs encoding the variable regions of mDG1 were cloned into plasmids containing sequences encoding constant regions of IgE heavy and kappa light chains in order to produce full-length IgE. HEK293 cells were co-transfected with the two expression vectors to transiently express a chimeric human IgE (chIgE-DG1) harboring the mDG1 paratope. After transfection, expression of chIgE-DG1 in the culture supernatant was assayed by Western blotting with anti-human Fcε (anti-hFcε) and anti-human κ light chain (anti-hkL) antibodies. A single band was revealed at approximately 190 kDa with both anti-hFcε and anti-hkL antibodies. After reduction, this band shifted to two bands, resolving at approximately 70 kDa with anti-hFcε and 25 kDa with anti-hkL (Fig 4A), as expected due to the formation of disulfide bridges between the two heavy chains of 70 kDa and two light chains of 25 kDa. The activity and specificity of chIgE-DG1 and mDG1 were compared via indirect ELISA on native gliadins and D-GLIA 48. As expected, no reactivity on native gliadins was observed. ChIgE-DG1 and mDG1 displayed equivalent titration curves for the detection of deamidated gliadins independently of the isotype (Fig 4B).

### ChIgE-DG1 sensitization allowed basophil degranulation

**Fig 5:**
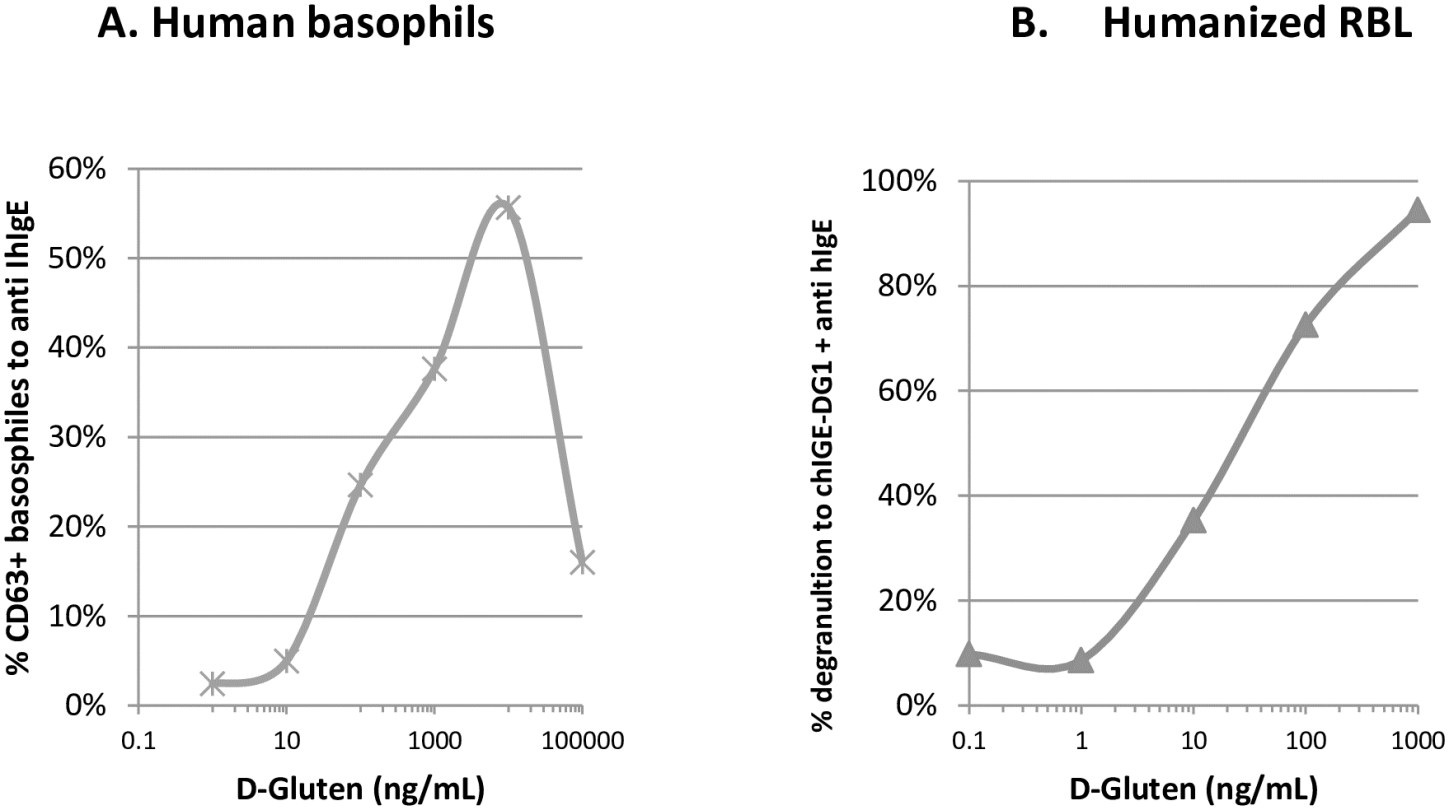
Chimeric mouse/human IgE-DG1 functionality on human basophils. A. Basophils from 2 human donors were passively sensitized with chIgE-DG1 and subsequently cross-linked with anti-human IgE antibodies or 6 doses of D-Gluten. For each D-Gluten dose, the mean percentage of CD63^+^ cells was monitored by flow cytometry and compared to the percentage of CD63^+^ cells induced by anti-human IgE. B. D-Gluten, Deamidated Gluten.

The ability of chIgE-DG1 to bind to cells expressing the human FcεRI receptor was verified on human basophils and on RBL-SX38 cells. Basophils from human donors were stripped and passively sensitized with chIgE-DG1 before stimulation with either anti-human IgE or D-Gluten. Crosslinking of chIgE-DG1 with anti-human IgE induced basophil degranulation demonstrating that chIgE-DG1 is biologically functional. More interestingly, basophils sensitized with the single chIgE-DG1 antibody were able to degranulate in a dose-dependent manner when triggered with D-Gluten. Maximum degranulation was reached with D-Gluten at 10 μg/mL and the EC_50_ was graphically estimated at 200 ng/mL (Fig 5). Then, RBL-SX38 cells were also passively sensitized with chIgE-DG1 at 100 ng/mL. As observed with human basophils, D-Gluten was able to induce the degranulation of chIgE-DG1-sensitized RBL-SX38 cells. Maximum degranulation was almost reached at 1000 ng/mL, and the EC_50_ was graphically estimated to be between 20 and 30 ng/mL (Fig 5).

### Comparison of chIgE-DG1 reactivity with sera from acid-HWP-allergic patients

**Fig 6:**
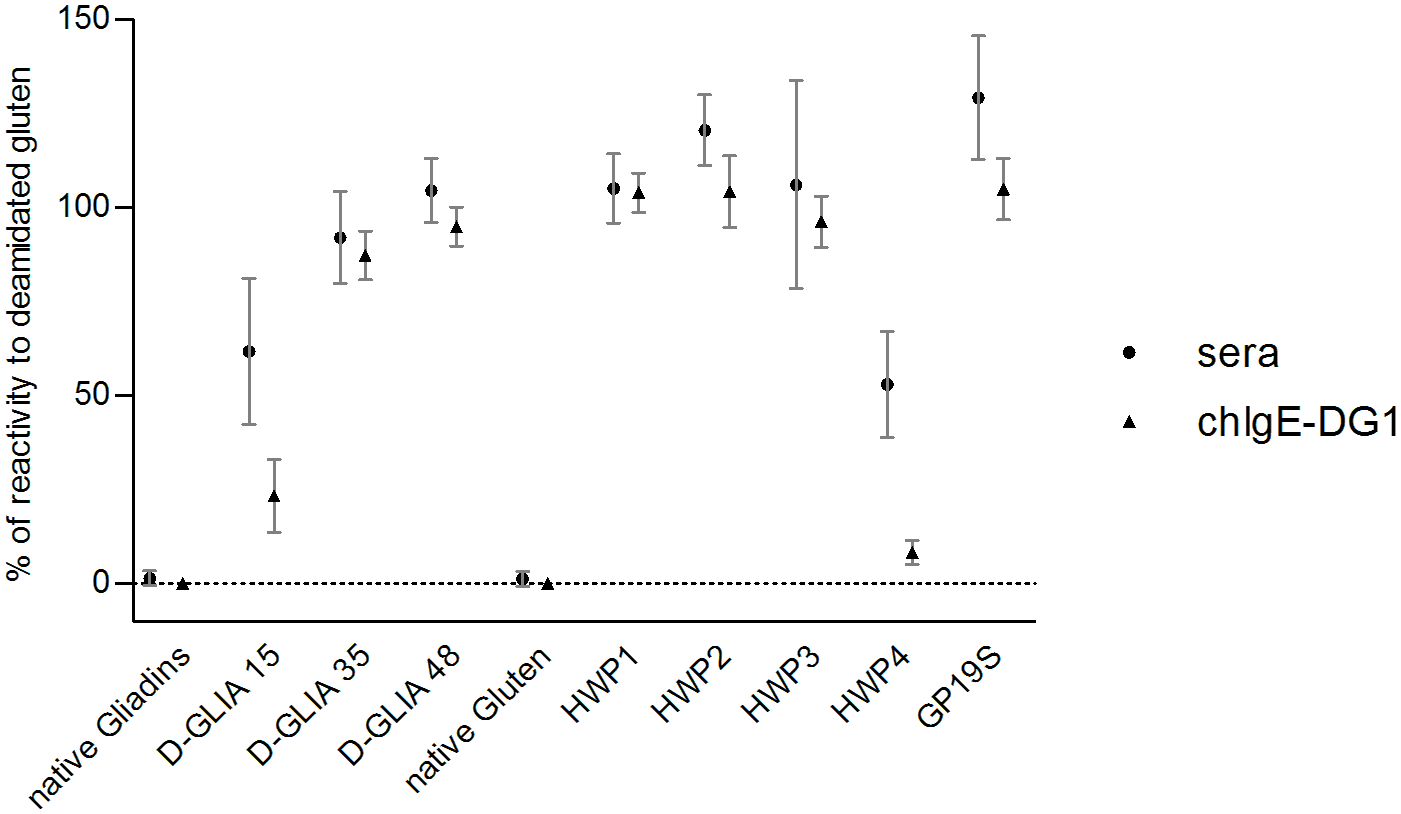
Reactivity of chimeric IgE-DG1 to various deamidated wheat protein fractions and comparison with patients' reactivity. The concentrations of chIgE-DG1 bound to native gliadins; native gluten; deamidated gliadins (D-GLIA15, 35, 48); D-gluten; HWP1, 2, 3, 4; and GP19S were determined by indirect ELISA. The results were normalized by determining the ratio of IgE for each protein to specific IgE for D-gluten. Symbols represent means of the ratio for human sera (circle, n = 9) or chIgE-DG1 (triangle, n = 3). Error bars represent ± SD. HWP, hydrolyzed wheat protein. GP19S, Glupearl 19S^®^.

ChIgE-DG1 was then applied on our set of deamidated gliadins and HWPs, and its reactivity profile was compared with the reactivity profiles of sera from acid-HWP-allergic patients. ChIgE-DG1 bound to all deamidated samples, with no reactivity to native gliadins and gluten (Fig 6). Specific IgE ratios were determined using D-Gluten as the reference and compared with the means of IgE ratios already determined from human sera (plotted as stars). All of the samples, except for D-GLIA 15 and HWP4, exhibited similar binding between chIgE-DG1 and patients' sera. For D-GLIA 15 and HWP4, chIgE-DG1 binding was notably lower than that with human IgE.

**Fig 7:**
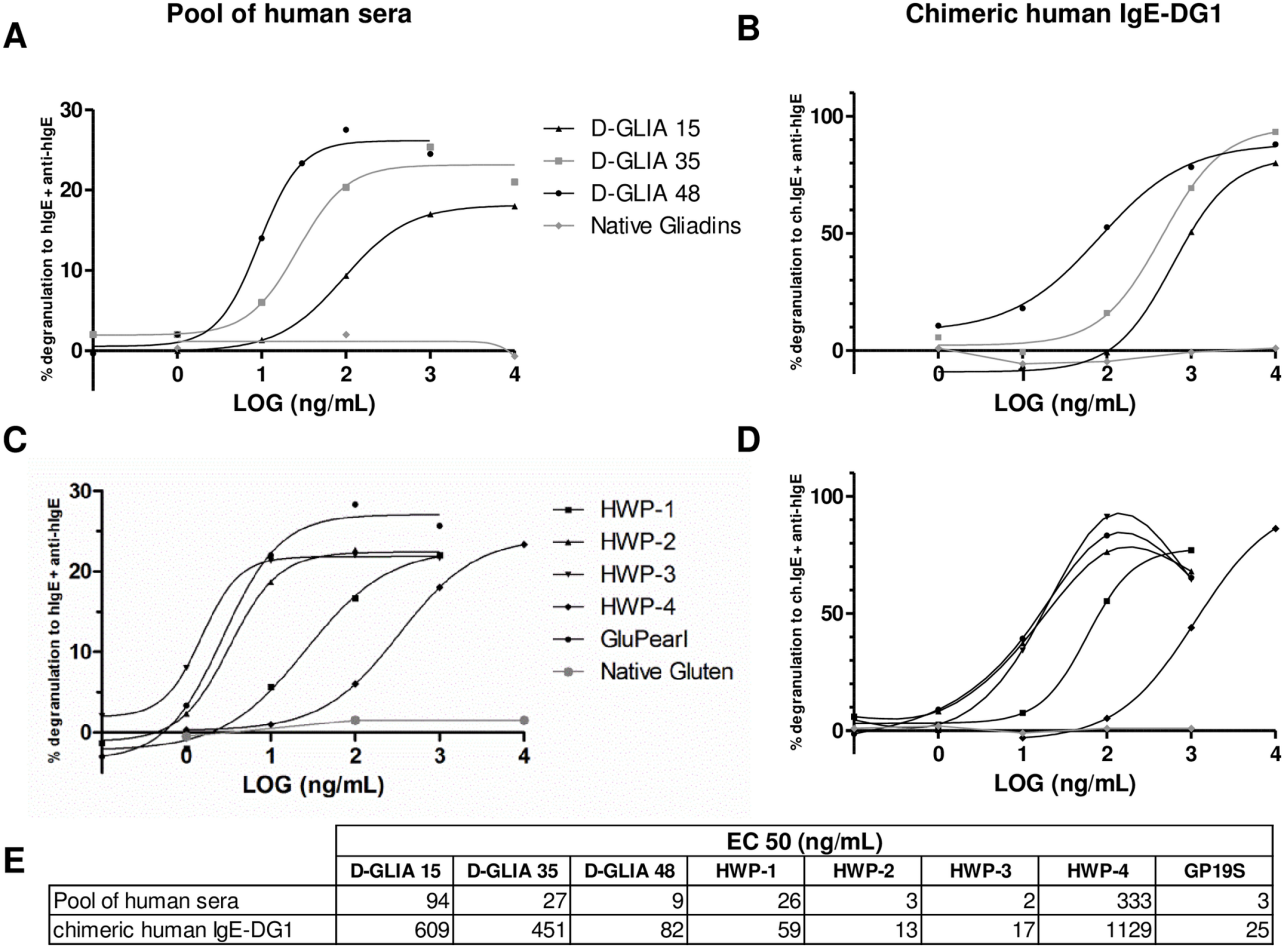
Comparison of human IgE and chimeric IgE-DG1 in an RBL SX38 assay with deamidated gliadins and HWP. RBL SX38 cells were sensitized with either a pool of 6 human sera (1/25; A and C) or chimeric IgE-DG1 at 100 ng/mL (B and D) and subsequently cross-linked with anti-human IgE antibody or five concentrations (1 ng/mL to 10 μg/mL) of native and deamidated gliadins (A and B); native gluten; HWP1, 2, 3, 4; or GP19S (C and D). The results are expressed as the percentage of degranulation compared to anti-human IgE-induced degranulation. The results corresponded to the mean of 3 independent experiments. EC_50_ values were determined by the regression of 4 parameters (E). D-GLIA, deamidated gliadin. HWP, hydrolyzed wheat protein. GP19S, Glupearl 19S^®^.

The functionality of the chIgE-DG1 antibody was compared to that of the pool of the 6 positive patients’ sera (sera #30, #285, #299, #352, #1414 and #1649) in the RBL-SX38 degranulation assay. RBL-SX38 cells were passively sensitized with either the pool of sera or with chIgE-DG1 and stimulated with serially diluted native or deamidated samples. Native gliadins and gluten did not induce cell degranulation, whereas all deamidated samples induced β-hexosaminidase release of the cells sensitized either with the serum pool or with chIgE-DG1 (Fig 7). All HWPs elicited a similar maximal degranulation when cells were sensitized with the pool of sera or with chIgE-DG1 (22–28% and 76–91%, respectively). The triggering capacity of the various deamidated samples was compared by determining their EC_50_ values in the two sensitizing conditions (table in Fig 7). The industrial samples HWP2, 3 and GP19S displayed the lowest EC_50_, resulting in the highest and equivalent triggering potential. HWP1 and 4 showed intermediate and low triggering potential, respectively. Although EC_50_ values obtained when cells were sensitized with the pool of sera or with the chIgE-DG1 were not in the same order magnitude, the ranking of the samples was the same and the gaps between degranulation curves were comparable for chIgE-DG1 and the pool of sera.

## Discussion

Acid-HWPs have been shown to trigger numerous cases of contact or food allergies in Europe and Japan. *In vivo* models have been used to investigate the allergenic potency of different HWPs in comparison to native gluten proteins [28,33,34]. However, animal models pose ethical considerations and are not well suited for screening a large number of samples. In this study, we took advantage of the known IgE-binding epitopes involved in allergic reactions to acid-HWPs and developed a chimeric recombinant IgE usable as an *in vitro* evaluation tool of the antigenic and triggering potency of functionalized glutens. The effect of the deamidation level of the products on the reactivity of IgEs from patients allergic to acid-HWP was first explored. Then, patients' and chimeric IgE responses were compared via ELISA and a functional basophil degranulation assay.

A set of laboratory and industrial deamidated wheat proteins were used in the study. According to analysis, the samples could be classified and ranked into 3 groups, characterized by their weak (approximately 15%), medium (approximately 30%) or high (50–60%) level of deamidation

IgEs from HWP-allergic patients reacted with all deamidated industrial and laboratory samples, while they did not bind or bound only very weakly to the native gliadin or gluten samples. They reacted markedly more strongly with the samples with high and medium deamidation rates than with the weakly modified samples. Variability in IgE binding among patients’ sera was sample dependent; it was particularly high for D-GLIA 15 and HWP3 and 4. For HWP3, this variability may be linked to the heterogeneity of the composition of this product, which is both deamidated and extensively hydrolyzed.

The products with medium and high deamidation rates also possessed the strongest ability to activate RBL-SX38 cells sensitized with patient IgE. In addition, mAb mDG1, presenting a restricted reactivity to the heptapeptides E/QPEEPFPE, was able to markedly inhibit the activation of HWP-induced RBL cells. These results highlight that the allergenicity of deamidated products was mainly borne by epitopes with a relatively high modification level.

These results are in accordance with the sequential epitopes identified in our previous work [11]. Using synthetic peptides corresponding to a consensus IgE-binding sequence with all combinations of glutamine substitutions into glutamic acid, we observed increasing IgE binding with increasing deamidation rate, and the QPEEPFPE sequence showed the highest IgE-binding intensity. However, IgE binding to weakly deamidated sequences was also observed. Although these responses occurred with a low binding intensity and with variability among patients, they might explain why inhibition of degranulation by mDG1 was incomplete. Our previous studies indicate that human sera recognized a broader repertoire of peptides than did mAb mDG1 [11,13]. In contrast to their reaction to this mAb, most patients’ sera were still able to react with peptides having only one or two Q/E substitutions and tolerated the presence of glutamine at positions Q_2_, Q_3_ or Q_4_.

Due to the significant contribution to IgE-binding and basophil activation of the deamidated epitopes bound by mDG1, this mAb was used to generate a chimeric mouse/human recombinant IgE specific for these sequences. The mAb was produced in the HEK293 cell line using an expression vector obtained by inserting cDNA encoding variable domains of mAb mDG1 into plasmids containing the sequences encoding the human IgE scaffold according to Christensen et al. (2008). The resulting recombinant chIgE-DG1 retained the activity and the specificity of the original mouse IgG and was able to bind to the human FcεRI receptor on both human basophils and RBL-SX38 cells. More interestingly, human basophils and RBL-SX38 cells sensitized with this unique chIgE-DG1 were activated by deamidated gluten. This ability of deamidated gluten to induce the degranulation of basophils sensitized with a single monoclonal IgE has to be related to the repetition of the mAb epitopes within γ and ω2-gliadin sequences. The repetition of linear epitopes in the allergen sequence is a distinctive feature of the repeated domains of gluten proteins [35,36]. We previously hypothesized that in ω-gliadins, composed of a unique repeated domain, these recurrent epitopic sequences may promote efficient effector cell degranulation [11,37]. In the case of deamidated wheat proteins, using a monoclonal IgE, we proved here that repeated epitopes were able to trigger basophil activation and therefore strongly contributed to gluten protein allergenicity. However, this feature may not be a general attribute of all gluten proteins, as α-gliadin repetitive domain alone was not able to induce RBL cell activation [38]. Recently, Gieras et al. showed that both the number and the proximity of epitopes on the sequence influenced effector cell activation capacity. Artificial allergens containing four IgE epitopes in adjacent positions were more potent at inducing basophil degranulation than molecules with fewer epitopes or composed of distantly placed epitopes [24]. Deamidated γ and ω2-gliadins are the main allergens involved in allergy to acid-HWPs [11]. These allergens appeared to fulfill both criteria; they not only contain a large number of repetitions (8/9) of the consensus IgE-binding epitope but these sequences also are in close proximity to each other.

We established that the chIgE-DG1 possesses an IgE-binding capacity equivalent to that of patients’ sera on laboratory and industrial samples with medium and high deamidation rates and did not react with native gliadin and gluten fractions. As expected, because the reactivity of its paratope is limited to a few highly deamidated epitopes, the chIgE-DG1 possessed a more restricted specificity than did most of the patients’ sera.

The chIgE-DG1 also mimicked patient sera when used in the functional RBL assay. In this assay, the various deamidated products were ranked in the same order according to their triggering potential, irrespective of the sensitizing antibodies (chIgE-DG1 or human IgE pool). Regarding the five industrial products, the products with the highest potency were the same in the two sensitizing conditions, i.e., HWP2, 3 and GP19S. The HWP1 product displayed an intermediate response; depending on the antibodies, it was 3 to 10 times less potent for cell activation than the former products; HWP4 was less active, approximately 100 times less active than HWP2. The ranking of products in the RBL assay matched the classification performed on the basis of the deamidation levels. Although HWP3 presented a higher degree of proteolysis than HWP2, this did not affect its triggering potential.

Functionalized glutens are among ingredients derived from plant proteins usable as animal protein substitutes in the context of sustainable food production. Safety assessment of modified glutens is therefore required with respect to the significant allergenic risk of acid-HWPs, which has been highlighted by the numerous Japanese cases and by the severity of reactions in both Europe and Japan. Although, no cases of allergy to acid-HWPs have been reported by the French Allergy Vigilance Network from 2014, Nakamura recently showed that such ingredients were still released on the market by European companies [9]. Human sera are not available in sufficient amounts for risk assessment purposes. In addition, highly specific IgE concentrations are generally needed in effector cell activation tests. The basophil activation test based on RBL-SX38 cells sensitized with chIgE-DG1 therefore appeared to be a suitable *in vitro* alternative to the use of human sera or animal models. This test can be used to assess the allergenicity of functionalized wheat proteins. In the same way, Knipping et al. recently proposed an RBL degranulation assay based on a pool of β-lactoglobulin-specific chimeric IgE for the *in vitro* assessment of whey hydrolysate [25]. A ring-trial for intra- and inter-laboratory validation showed that this type of assay based on humanized RBL cells and chimeric mouse/human IgE monoclonal antibodies was a robust, reproducible, and promising tool for use in safety assessment [39].

Of note, for the group of French sera evaluated in this study, the HWP GP19S commercialized in Japan displayed IgE-binding and triggering capacities matching those of two products released in Europe. Although the sensitization mode and the duration of exposition differed in these populations, cases of allergy to acid-HWPs presented high similarities, notably in the type and severity of symptoms. The larger number of cases in Japan probably resulted from a combination of factors such as the wide exposure to the GP19S-containing soap, the presence of a detergent that was shown to favor sensitization by gluten proteins [34], and repeated skin applications [40]. Similar dominant epitopes and allergens have also been reported for both patient populations [11,12]. We confirmed here that the culprit allergen sources possessed similar biochemical and allergenic properties. These results suggest that French and Japanese patients suffered from the same unconventional allergy to deamidated wheat proteins generated by acid hydrolysis.

The comparison between patients’ and chimeric IgE antibodies enables investigations of the composition of patients' IgE repertoire. The similar reactivity of patients’ IgEs and of the chimeric anti-peptide IgE suggests that continuous epitopes play an important role in this type of allergy. The equivalent triggering activity of the IgE pool with that of the monoclonal chIgE-DG1 suggests that a limited IgE repertoire might be involved in allergy to acid-HWPs, with IgE preferentially binding highly deamidated sequences. However, we observed that the patients’ sera displayed a somewhat larger reactivity than the chIgE-DG1, notably towards low deamidated products or epitopes [11,13]. Chemical deamidation is a random process. Within the glutamine-rich repeat domains of gluten proteins, this process randomly generates discrete sequences with various deamidation levels (Tranquet 2015), with the amount of highly deamidated sequences increasing with the treatment intensity. It is not easy to clarify whether the broader patient reactivity results from a restricted repertoire of IgEs that are able to cross-react with sequences possessing various combinations of deamidation or whether it stems from a larger IgE repertoire.

To conclude, a chimeric mouse/human IgE was produced against the deamidated epitope QPEEPFPE involved in allergy to acid-HWP products. This recombinant antibody was equivalent to patient sera in its ability to classify and rank industrial deamidated glutens in a basophil activation assay and could be a relevant tool in the allergenicity evaluation of functionalized glutens. This reagent also demonstrates that epitope organization in the repeated domains of some gluten proteins allows a single monoclonal IgE to efficiently trigger effector cells. This study also shows that the IgE repertoire of French acid-HWP-allergic patients might be restricted and that effector cells are mainly activated by highly deamidated epitopes.

## Supporting information

S1 Fig SDS Page analysis of Hydrolyzed wheat proteins (HWP) on 4–20%. Hydrolyzed wheat protein (30μg) or native gluten (10 μg) were separeated on StainFree 4–20% gels (Bio-Rad). Proteins were revealed by 5 min. photocativation of in gel-flurorochrome which bind to tryptophan (GelDoc EZ system, BioRad). GP, Glupearl19S. Glut, native gluten. D-Glut, deamidated gluten.
(DOCX)

S1 Table Clinical and serological characteristics of allergic patients. Patient’s reactivity towards native proteins (Wheat or Gluten) or Deamidated Gluten (D-Gluten from ALK-Abello) was tested by skin prick test. Specific IgE concentrations to albumins/globulins fraction (A/G), gliadins, ω5 gliadins, LTP and D-Gluten (laboratory made) were determined by F-ELISA.–negative; nd, not determined; U, Urticaria; EIA, Exercise induced anaphylaxis; AS, Anaphylaxis; AD Atopic dermatitis.
(DOCX)

S2 Table Specific IgE concentrations of allergic patients to various native and deamidated wheat protein fractions. Concentrations of total and specific IgE were determined by F- ELISA. D-GLIA, Deamidated Gliadins. HWP, Hydrolyzed Wheat Proteins. GP19S, GluPearl 19S^®^.
(DOCX)
