# Supplementary material for: A chimeric IgE that mimics IgE from patients allergic to acid-hydrolyzed wheat proteins is a novel tool for in vitro allergenicity assessment of functionalized glutens
Source: PLoS One. 2017 Nov 8;12(11):e0187415. doi: 10.1371/journal.pone.0187415 (PMC5678878; doi:10.1371/journal.pone.0187415)
Supplement: S1 Fig — Hydrolyzed wheat protein (30μg) or native gluten (10 μg) were separeated on StainFree 4–20% gels (Bio-Rad). Proteins were revealed by 5 min. photocativation of in gel-flurorochrome which bind to tryptophan (GelDoc EZ system, BioRad). GP, Glupearl19S. Glut, native gluten. D-Glut, deamidated gluten. (DOCX) [file pone.0187415.s001.docx]

**S1 Fig. SDS Page analysis of Hydrolyzed wheat Proteins (HWP) on 4-20%**

Hydrolyzed wheat protein (30µg) or native gluten (10 µg) were separeated on StainFree 4-20 % gels (Bio-Rad). Proteins were revealed by 5 min. photocativation of in gel-flurorochrome which bind to tryptophan (GelDoc EZ system, BioRad). GP, GluPearl19S. Glut, native gluten. D-Glut, deamidated gluten.


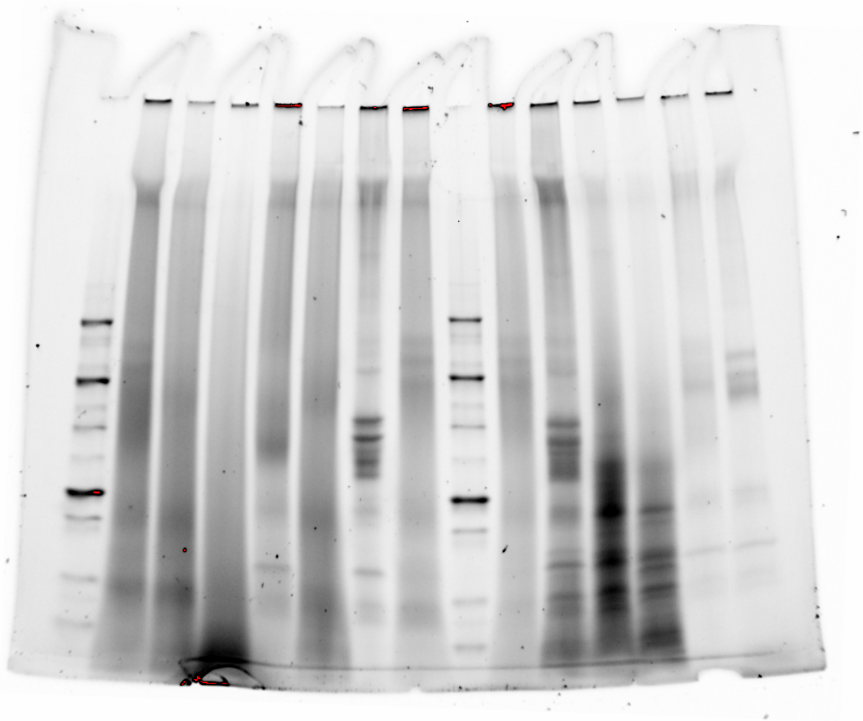


75

50

35

25

20

15

10

HWP PM

1 2 3 4 GP Glut D-Glut
