# Supplementary material for: A chimeric IgE that mimics IgE from patients allergic to acid-hydrolyzed wheat proteins is a novel tool for in vitro allergenicity assessment of functionalized glutens
Source: PLoS One. 2017 Nov 8;12(11):e0187415. doi: 10.1371/journal.pone.0187415 (PMC5678878; doi:10.1371/journal.pone.0187415)
Supplement: S1 Table — Patient’s reactivity towards native proteins (Wheat or Gluten) or Deamidated Gluten (D-Gluten from ALK-Abello) was tested by skin prick test. Specific IgE concentrations to albumins/globulins fraction (A/G), gliadins, ω5 gliadins, LTP and D-Gluten (laboratory made) were determined by F-ELISA.–negative; nd, not determined; U, Urticaria; EIA, Exercise induced anaphylaxis; AS, Anaphylaxis; AD Atopic dermatitis. (DOCX) [file pone.0187415.s002.docx]

**S2 Table. Clinical and serological characteristics of allergic patients**

Patients reactivity towards native proteins (Wheat or Gluten) or Deamidated Gluten (D-Gluten from ALK-Abello) was tested by skin prick test. Specific IgE concentrations to albumins/globulins fraction (A/G), gliadins, ω5 gliadins, LTP and D-Gluten (laboratory made) were determined by F-ELISA. – negative; nd, not determined; U, Urticaria; EIA, Exercise induced anaphylaxis; AS, Anaphylaxis; AD Atopic dermatitis.

|  | Patients’ information | | | | Skin Prick test (mm) | | | Specific IgE concentration (ng/mL) | | | | |
| --- | --- | --- | --- | --- | --- | --- | --- | --- | --- | --- | --- | --- |
|  | Sera | Age | Sexe | Symptoms | Wheat | Gluten | D-Gluten | A/G | Gliadins | ω5 | LTP | D-Gluten |
| Allergic to acid-HWP | # 30^*^ | 24 | F | U, U | - | 2.5 | 7 | 5 | 17 | 13 | 0 | 174 |
|  | # 34^*^ | 18 | F | EIA, U | - | - | 8.5 | 0 | + | 0 | 0 | 34 |
|  | # 285^*^ | 10 | F | AS | - | nd | 6 | nd | nd | nd | nd | 142 |
|  | # 299^*^ | 8 | M | AS | - | nd | 5.5 | 0 | + | + | nd | 119 |
|  | # 352^*^ | 12 | M | EIA | - | - | 4.5 | 0 | + | 0 | 0 | 216 |
|  | # 390^*^ | 10 | M | U | - | 3 | 5 | 0 | 4 | 0 | 0 | 60 |
|  | # 833 | 28 | F | AS | nd | nd | 5 | nd | 0 | 0 | nd | 23 |
|  | # 1414 | 15 | M | EIA | 3.5 | 7 | 5 | 0 | 0 | 0 | 0 | 122 |
|  | # 1649 | 14 | F | AS | - | nd | 10 | 5 | 5 | 0 | 0 | 229 |
| Wheat | #1274 | 8 | M | AD | 7 | 6 | 6 | 98 | 89 | 0 | 30 | 0 |

- Sera already presented in Denery et al allergy, 2012
