# Supplementary material for: A chimeric IgE that mimics IgE from patients allergic to acid-hydrolyzed wheat proteins is a novel tool for in vitro allergenicity assessment of functionalized glutens
Source: PLoS One. 2017 Nov 8;12(11):e0187415. doi: 10.1371/journal.pone.0187415 (PMC5678878; doi:10.1371/journal.pone.0187415)
Supplement: S2 Table — Concentrations of total and specific IgE were determined by F- ELISA. D-GLIA, Deamidated Gliadins. HWP, Hydrolyzed Wheat Proteins. GP19S, GluPearl 19S®. (DOCX) [file pone.0187415.s003.docx]

**S3 Table. Specific IgE concentrations of allergic patients to various native and deamidated wheat protein fractions**

Concentrations of total and specific IgE were determined by F- ELISA. D-GLIA, Deamidated Gliadins. HWP, Hydrolyzed Wheat Proteins. GP19S, GluPearl 19S^®^.

|  | IgE concentration - ng/ml | | | | | | | | | | | |
| --- | --- | --- | --- | --- | --- | --- | --- | --- | --- | --- | --- | --- |
| Sera | Total IgE | native Gliadins | D-GLIA 15 | D-GLIA 35 | D-GLIA 48 | Deam. Gluten | native Gluten | HWP1 | HWP2 | HWP3 | HWP4 | GP19S |
| # 30^*^ | 998 | 0 | 139 | 209 | 182 | 174 | 0 | 196 | 202 | 189 | 106 | 232 |
| # 34^*^ | 475 | 0 | 20 | 34 | 40 | 34 | 0 | 41 | 45 | 47 | 20 | 50 |
| # 285^*^ | 2569 | 6 | 124 | 127 | 168 | 142 | 6 | 150 | 178 | 209 | 97 | 194 |
| # 299^*^ | 520 | 0 | 47 | 101 | 129 | 119 | 0 | 109 | 157 | 143 | 41 | 166 |
| # 352^*^ | 928 | 4 | 143 | 196 | 214 | 216 | 4 | 213 | 259 | 199 | 127 | 247 |
| # 390^*^ | 631 | 0 | 44 | 56 | 58 | 60 | 0 | 61 | 62 | 55 | 35 | 60 |
| # 833 | 181 | 0 | 6 | 21 | 24 | 23 | 0 | 26 | 26 | 26 | 6 | 29 |
| # 1414 | 231 | 6 | 72 | 103 | 114 | 122 | 6 | 116 | 139 | 69 | 61 | 141 |
| # 1649 | 895 | 6 | 157 | 172 | 224 | 229 | 0 | 253 | 297 | 200 | 143 | 348 |
| #1274 | 22451 | 88 | 41 | 34 | 25 | 0 | 0 | 4 | 0 | 0 | 4 | 0 |

- Sera already presented in Denery et al allergy, 2012
